# Age‐dependent, negative heterozygosity–fitness correlations and local effects in an endangered Caribbean reptile, *Iguana delicatissima*

**DOI:** 10.1002/ece3.3826

**Published:** 2018-01-18

**Authors:** Jessica L. Martin Judson, Charles R. Knapp, Mark E. Welch

**Affiliations:** ^1^ Department of Biological Sciences Mississippi State University Mississippi State MS USA; ^2^ San Diego Zoo Institute for Conservation Research Escondido CA USA; ^3^ Present address: Daniel P. Haerter Center for Conservation and Research John G. Shedd Aquarium Chicago IL USA

**Keywords:** conservation, heterozygosity, inbreeding, Lesser Antillean Iguana

## Abstract

Inbreeding depression can have alarming impacts on threatened species with small population sizes. Assessing inbreeding has therefore become an important focus of conservation research. In this study, heterozygosity–fitness correlations (HFCs) were measured by genotyping 7 loci in 83 adult and 184 hatchling Lesser Antillean Iguanas, *Iguana delicatissima,* at a communal nesting site in Dominica to assess the role of inbreeding depression on hatchling fitness and recruitment to the adult population in this endangered species. We found insignificant correlations between multilocus heterozygosity and multiple fitness proxies in hatchlings and adults. Further, multilocus heterozygosity did not differ significantly between hatchlings and adults, which suggests that the survivorship of homozygous hatchlings does not differ markedly from that of their heterozygous counterparts. However, genotypes at two individual loci were correlated with hatching date, a finding consistent with the linkage between specific marker loci and segregating deleterious recessive alleles. These results provide only modest evidence that inbreeding depression influences the population dynamics of *I. delicatissima* on Dominica.

## 1 INTRODUCTION

Inbreeding depression has long been a concern for small populations facing the threat of extinction. During population bottlenecks, inbreeding increases, and the frequencies of deleterious recessive alleles increase due to the enhanced role of genetic drift (Hedrick & Kalinowski, 2000). Small populations with increased inbreeding rates may be less able to adapt to changes in the environment caused by human disturbance or climate change, which can further decrease population sizes and increase the severity of inbreeding depression (Armbruster & Reed, 2005). Close management of inbreeding prevalence in imperiled species is often desired to prevent increased population declines, but monitoring inbreeding in the wild can be a significant challenge (Grueber, Waters, & Jamieson, 2011; Hansson & Westerberg, 2002; Kruuk & Hill, 2008). Thus, inbreeding is frequently measured indirectly through heterozygosity–fitness correlations (HFCs) that contrast heterozygosity at multiple nuclear loci with measures of fitness (Chapman, Nakagawa, Coltman, Slate, & Sheldon, 2009; Coltman & Slate, 2003; Miller & Coltman, 2014).

Many studies report significant positive HFCs (reviewed in Hansson & Westerberg, 2002; in reptiles, Shaner, Chen, Lin, Kolbe, & Lin, 2013; Phillips, Jorgenson, Jolliffe, & Richardson, 2017). However, the hypothesized reasons for these correlations are varied, and not all HFCs lend insight into inbreeding (Chapman et al., 2009). Three prevailing hypotheses have been postulated to explain the relationship between multilocus heterozygosity and fitness. Under the direct effect hypothesis, loci under study impact the fitness of an individual and are directly responsible for the observed HFCs (Hansson & Westerberg, 2002). This hypothesis is commonly invoked in studies of allozymes, single‐nucleotide polymorphisms, or major histocompatibility complex loci, as homozygosity at these loci can potentially alter biochemical efficiency or physical traits in individuals (Grueber, Wallis, & Jamieson, 2008; Hansson & Westerberg, 2002). According to the local effect hypothesis, the correlation of heterozygosity at neutral loci (e.g., microsatellites) with fitness traits is due to linkage disequilibrium with loci directly affecting fitness (Grueber et al., 2008). Finally, the general effect hypothesis posits that heterozygosity at neutral marker loci is correlated with heterozygosity across the individual's genome, and a reduction in the fitness of homozygous individuals reflects inbreeding (Szulkin, Bierne, & David, 2010).

Although many microsatellite studies have detected HFCs in wild populations, most typically do not reveal strong HFCs, and the detected HFCs are usually only weakly significant (Chapman et al., 2009). Multiple explanations for this gap between theory and empirical evidence have been presented, including the use of large, essentially panmictic populations in which significant inbreeding depression is likely rare (Grueber et al., 2008). Chapman et al. (2009) have suggested that the use of fitness‐correlated traits that are only weakly influenced by inbreeding may also hamper detection of HFCs, as might the varied demographic history of populations under study (see also Canal, Serrano, & Potti, 2014; Miller et al. 2014). Further, using a small number of loci to calculate heterozygosity may fail to represent heterozygosity across the genome, which is a critical assumption of the inbreeding interpretation of HFCs (Miller & Coltman, 2014; but see Brommer, Kekkonen, & Wikstrom, 2015).

Age class is a frequently overlooked aspect of evaluating inbreeding depression in wild populations using HFC approaches. Natural selection has a strong effect on younger age classes, while its effects in later life stages may be less influential (Hamilton, 1966; Medawar, 1952). Therefore, younger age classes are expected to exhibit greater variation in fitness than older age classes, as unfit individuals likely will not survive to adulthood (Koehn & Gaffney, 1984). The population dynamics of aging cohorts may have a large effect on HFC detection if unfit genotypes are less common in the surviving individuals (David & Jarne, 1997), which may mask the true effects of inbreeding on fitness when only an older cohort is sampled. For example, Cohas, Bonenfant, Kempenaers, and Allainé (2009) detected inbreeding depression among juvenile alpine marmots, as multilocus heterozygosity (MLH) correlated strongly with juvenile survival. Among adult marmots, however, no correlation between MLH and fitness was revealed (Cohas et al., 2009). Recent studies have further emphasized the importance of assessing multiple age classes in the detection and direction of HFCs (Annavi et al., 2014; Brommer et al., 2015; Canal et al., 2014; Monceau, Wattier, Dechaume‐Moncharmont, Dubreuil, & Cezilly, 2013).

**Figure 1:**
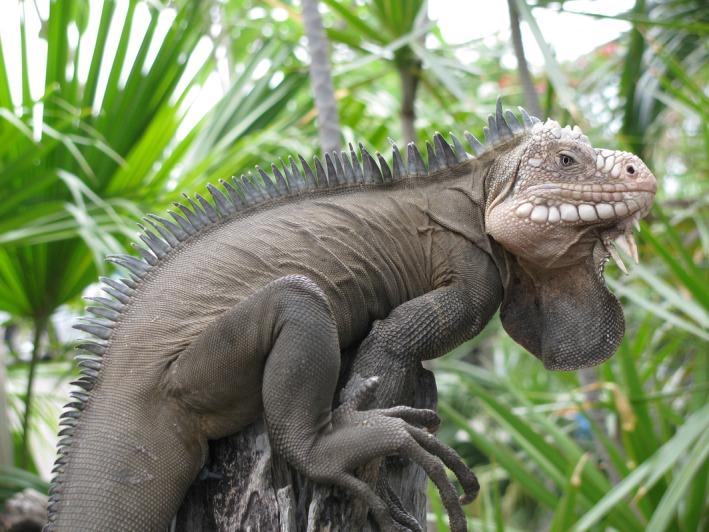
Male Lesser Antillean Iguana. Image of a male Lesser Antillean iguana taken by C. Knapp in the Commonwealth of Dominica

The Lesser Antillean Iguana (*Iguana delicatissima*) is a large terrestrial lizard native to the northern islands of the Lesser Antilles (Figure 1; Breuil, Day, & Knapp, 2010). Despite the large geographic range of *I. delicatissima* across the Lesser Antilles, the species is declining at an alarming rate and is now recognized as Endangered according to International Union for Conservation of Nature (IUCN) Red List criteria (Breuil et al., 2010). Not only is the species victim to many of the pressures facing other iguanid lizards in the Caribbean, such as predation by feral mammals and loss of habitat (Malhotra, Thorpe, Hypolite, & James, 2007), but it is also vulnerable to competition and hybridization with the invasive Green Iguana, *Iguana iguana* (Breuil et al., 2010; Martin, Knapp, Gerber, Thorpe, & Welch, 2015; Vuillaume, Valette, Lepais, Grandjean, & Breuil, 2015; van den Burg et al. in review). The Lesser Antillean Iguana plays a distinct ecosystem role as a seed disperser of coastal forest plants that are toxic to other vertebrates (Day, Breuil, & Reichling, 2000), and its further decline could impact the integrity of natural ecosystems.

The aims of this study were to assess whether age class is influential on the detection of HFCs in *I*. *delicatissima* and to evaluate whether adult heterozygosity is greater than in hatchlings due to selection against inbred, homozygous hatchlings. To accomplish these aims, we tested the hypothesis that inbreeding depresses fitness in *I. delicatissima* hatchlings and thus significantly reduces recruitment to adulthood. Indeed, inbreeding reduces hatching success, size, and survival to adulthood in other taxa (Coltman, Bowen, & Wright, 1998; Daniels & Walters, 2000; Stockley, Searle, Macdonald, & Jones, 1993). The beginning of life for iguanas is particularly challenging, as iguana hatchlings do not benefit from significant parental care and often must excavate a path out of their nest burrows after hatching (Bock & Rand, 1989). Further, predation rates of hatchlings are greater than that for adults due to their small size (Breuil et al., 2010). These factors create a stronger selective force on hatchlings with decreased fitness due to inbreeding, which may be magnified by the high density and competitive pressure of certain populations of *I. delicatissima*. We tested our hypothesis by calculating MLH of microsatellite markers to assess HFCs in three fitness‐related traits in both hatchling and adult iguanas in a population from the Commonwealth of Dominica. Further, we calculated the strength of selection on homozygous hatchling individuals through a comparison to adult homozygosity to assess the effect of inbreeding on recruitment. By comparing heterozygosities between the hatchling and adult age classes, this study not only allows insight into the effects of inbreeding on individual fitness, but also how inbreeding shapes survivorship and ultimately the dynamics of the adult population.

## 2 METHODS

### 2.1 Study area

**Figure 2:**
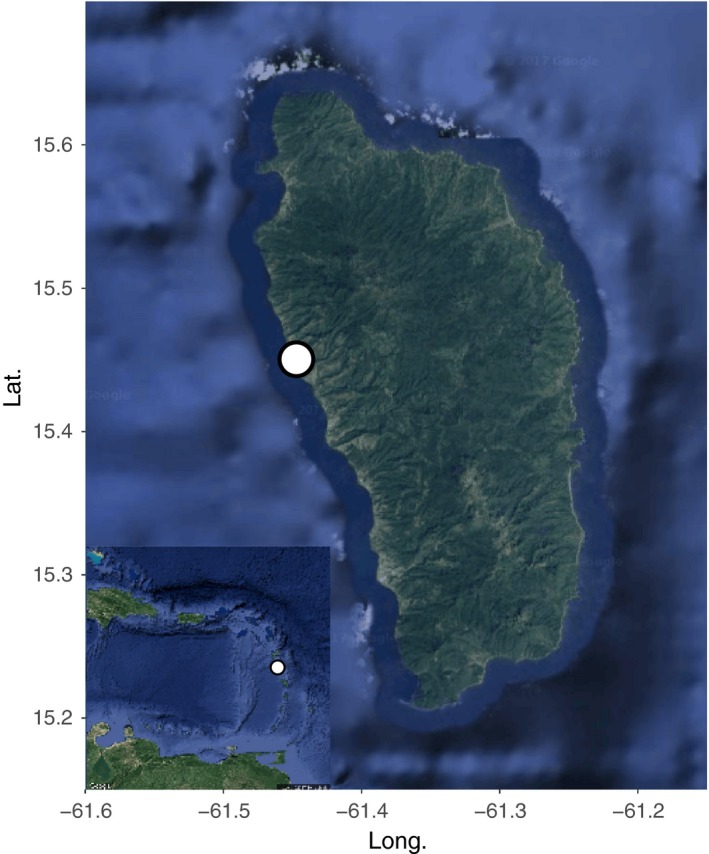
Satellite map of the Commonwealth of Dominica. A map of the island of Dominica in the Lesser Antilles. The sampling site at Batali Beach is marked. The inset map depicts the location of Dominica in the Lesser Antilles with Dominica marked. This map was created using the package ggmap (Kahle & Wickham, 2013) in R V. 3.3.1 (R Core Team 2016); map data ©2017 Google; ©2017 TerraMetrics

Dominica is one of the largest of the eastern Caribbean islands (48 km long and 24 km wide) with a maximum altitude of 1,447 m. The island is believed to support the largest population of *I. delicatissima* due to the extent of available coastal habitat. This study was conducted on the Caribbean (leeward) coast slope (350 m in length) at Batali Beach, located south of the Batali River (Figure 2). Female iguanas converge on the slope during the nesting season but concentrate most of their nesting in one communal area (Knapp, Prince, & James, 2016), thus allowing us to compare two age classes from the same region with minimal bias from underlying population genetic structure.

### 2.2 Sample collection

We captured free‐ranging adult iguanas by noose in the summers of 2007, 2008, and 2009. Earlier summer months coincided with the timing of female migration to communal nesting sites, whereas August and September aligned with the primary hatching period (Knapp & Perez‐Heydrich, 2012). Blood was collected by venipuncture of the ventral coccygeal vein using a heparinized syringe and stored in vacutainer tubes with 100 mM Tris, 100 mM Na_2_ EDTA, 10 mM NaCl, and 1% SDS at a ratio of 1:2 (Longmire, Maltbie, & Baker, 1997). Blood samples were stored at 4°C or −80°C. We also determined sex by cloacal probing for hemipenes and recorded morphometrics such as body mass (BM), snout‐vent length (SVL), and head width (HW). Iguanas in this study were considered adults at ≥25.0 cm SVL based on the smallest female confirmed gravid during the study (Knapp et al., 2016). Hatchlings were collected in August and September of 2009 from an enclosure built around the nesting site. We sampled hatchlings across days and across times during the day to minimize relatedness. BM, SVL, and hatching date were recorded, and blood was collected and stored in the same manner as adults. In total, 100 adult blood samples and 192 hatchling blood samples were used for this study.

### 2.3 Data collection

DNA was extracted from blood samples using either an ABI PRISMᵀᴹ 6100 Nucleic Acid Prep Station (Applied Biosystems, Foster City, California, USA) or a Maxwell® 16 Nucleic Acid Extraction System with a Maxwell® 16 Tissue DNA Purification Kit (Promegaᵀᴹ). To identify polymorphic microsatellite loci in *I. delicatissima*, 60 anonymous microsatellites identified in studies of *Cyclura* species (An et al., 2004; Lau et al., 2009; Rosas et al., 2008; Welch et al., 2011) were tested for amplification using a basic PCR touchdown cycle (Don, Cox, Wainwright, Baker, & Mattick, 1991). Sequence amplification was verified on 1% agarose gels. Of the 60 loci tested, 46 amplified successfully in *I. delicatissima*. M13 forward primer (CACGACGTTGTAAAACGAC) (Schuelke, 2000) labeled with one of four fluorescent dyes (FAM, NED, HEX, VIC) was then added to amplified loci for genotyping. Of these 46 loci screened for variation, nine loci were polymorphic in *I. delicatissima*. Samples were subsequently genotyped at these nine polymorphic loci, and genotype data were scored using Peak Scanner Version 1.0 (Applied Biosystems). Individuals scored at fewer than six microsatellite loci were excluded from further analyses.

### 2.4 Analyses

The program MICRO‐CHECKER (Van Oosterhout, Hutchinson, Wills, & Shipley, 2004) was used to identify loci with potential genotyping errors. Loci with a high probability of containing null alleles or scoring errors were excluded from further analyses to avoid inflating our estimates of inbreeding. After microsatellites were assessed for error, Arlequin v. 3.5.2.2 (Excoffier & Lischer, 2010) was used to estimate the inbreeding coefficient (*F*_*IS*_), observed and expected heterozygosity, departures from Hardy–Weinberg equilibrium, and allele frequencies for the adult and hatchling groups. The effective population size was calculated using adult individuals according to the linkage disequilibrium method of Waples (2006) as implemented in NeEstimator v. 2.01 (Do et al., 2014).

MLH was estimated according to three different approaches: standardized heterozygosity (SH; Coltman, Pilkington, Smith, & Pemberton, 1999), internal relatedness (IR; Amos et al., 2001), and heterozygosity by locus (HL; Aparicio, Ortego, & Cordero, 2006). Both IR and HL are inversely related to SH in that a positive correlation between heterozygosity and fitness is indicated by a negative correlation between IR or HL and the fitness trait (Aparicio et al., 2006). All HFC studies use at least one of these three MLH measures (Chapman et al., 2009; Grueber et al., 2011), and by including all three measures, our results can be more readily compared to other studies. Calculations for SH, IR, and HL were performed in the R (R Core Team 2016) package Rhh v. 1.0.2 (Alho, Välimäki, & Merilä, 2010). SH of adults and hatchlings was fitted to a normal curve, and the goodness of fit was calculated using a Shapiro–Wilk test to assess normality. The data were non‐normal (W = 0.98; *p *<* *.0003), and thus, we used a Wilcoxon signed‐rank test to evaluate differences in means between the hatchling and adult MLH measures (SH, IR, HL) and inbreeding coefficients (*F*_*IS*_) in JMP® v. 11.2.

For adults, we used BM, SVL, and HW as proxies of fitness, and in hatchlings, we used BM, SVL, and hatching date. Multiple studies have found a correlation between body size and fitness in lizards (Clobert et al., 2000; Le Galliard, Clobert, & Ferrière, 2004). In adult iguanas, body mass and SVL are positively correlated with dominance in males and clutch size in females (Alberts, Lemm, Perry, Morici, & Phillips, 2002; King, 2000; Knapp, Iverson, & Owens, 2006). Body mass and SVL are also deemed important fitness traits in hatchlings, as larger hatchlings tend to acquire resources and escape predators more successfully than their smaller counterparts in many lizard species (Clobert et al., 2000; Le Galliard et al., 2004). Head width was also included as a fitness variable for adults, as head width is correlated positively with bite force, which allows increased dominance and territory maintenance in male iguanas (Herrel, De Grauw, & Lemos‐Espinal, 2001). Further, head width is correlated with body size, and unlike mass, it is not affected by bias from seasonal variation associated with diet, or gravid state of females. Finally, hatching date was included as a fitness variable for hatchlings, as it may affect juvenile lizard survival, with the earliest‐hatching individuals experiencing increased growth rate and survival (Warner & Shine, 2007). Morphometric variables were standardized by sex (see Sokal & Rohlf, 1995) to minimize biases from sexual size dimorphism. Body mass of females captured in the nesting season was excluded from regression analysis as gravid females would skew this variable upward. All analyses were performed in JMP® v. 11.2 by regressing the fitness trait on each measure of MLH (SH, IR, and HL). Hatchling regressions were performed similarly, except traits were not standardized by sex. We used Holm's sequential Bonferroni adjustment to account for multiple comparisons (Holm, 1979).

We also distinguished between the causes of any HFCs, which are limited to general and local effects when using microsatellites. One requirement for general effects is that the heterozygosity of the marker loci must be correlated with heterozygosity across the genome, which is tested by calculating identity disequilibrium. Identity disequilibrium (ID) is the covariance in marker heterozygosity or the nonrandom heterozygosity association among loci in an individual (Miller & Coltman, 2014; Weir & Cockerham, 1969). ID was calculated using the g_2_ statistic in RMES (David, Pujol, Viard, Castella, & Goudet, 2007). The alternative hypothesis for HFCs in studies using microsatellites is the local effect hypothesis, in which the chosen markers are in linkage disequilibrium with loci that directly influence fitness. If certain microsatellite loci are indeed linked to these fitness loci, these microsatellites would more strongly correlate to the fitness trait in a simple regression than other loci and would be a greater contributor to the overall correlation between MLH and fitness. The data were tested for local effects using the method outlined in Szulkin et al. (2010):

(1) $$F=\frac{\left(resSS_{1}-\;resSS_{2}\right)/\left(df_{1}\;-\;df_{2}\right)}{resSS_{2}/df_{2}}$$

In this equation, *resSS*_1_ and *resSS*_2_ are the residual sums of squares for the simple regression using MLH and the multiple regression using all single‐locus heterozygosities, respectively. *df*_1_ and *df*_2_ are the degre of freedom for the two models. Szulkin et al. (2010) define their own MLH calculation as the sum of heterozygosity values across all loci, and a homozygous locus has a value of zero, while a heterogous locus has a value of one. This method compares the residual sums of squares of a simple regression of afitness trait on MLH to a multiple regression of a fitness trait on heterozygosity at each locus to test whether certain loci are contributing more heavily to observed HFCs than others. The resulting value is then compared to the critical F value with degrees of freedom (*df*_1_– *df*_2_, *df*_2_) (Szulkin et al., 2010). If the result from the equation is greater than the critical F value, then significant local effects are indicated. All traits for hatchlings and adults were tested for local effects using only individuals genotyped at all microsatellite loci. We again used Holm's sequential Bonferroni adjustment to account for multiple comparisons (Holm, 1979).

The intensity of selection was calculated using the MLH measures (SH, IR, and HL) of the hatchlings and adults according to Van Valen's (1965) alteration of Haldane's equation (Haldane, 1954):

(2) $$H=\frac{1}{2}\ln\left(\frac{S_{b}^{2}}{S_{a}^{2}}\right)+\frac{{\left({\overset{¯}{X}}_{a}-{\overset{¯}{X}}_{b}\right)}^{2}}{2(S_{b}^{2}-S_{a}^{2})}$$

(3) $$I=\;1-e^{-H}$$

where *S*_b_ is the standard deviation before selection, which in this case refers to the standard deviation in the MLH measure in the hatchlings, and *S*_a_ is the standard deviation after selection, which refers to the standard deviation of adult MLH. This equation relies on differences between heterozygosity of hatchlings and adults and uses these differences to determine the proportion of individuals that must be removed from the distribution of heterozygosity prior to selection to account for the observed difference in distribution after selection. The interpretation of this value for the intensity of selection relies on the assumption that all hatchling cohorts are similar in MLH means and variances to the cohort tested in this study.

## 3 RESULTS

**Figure 3:**
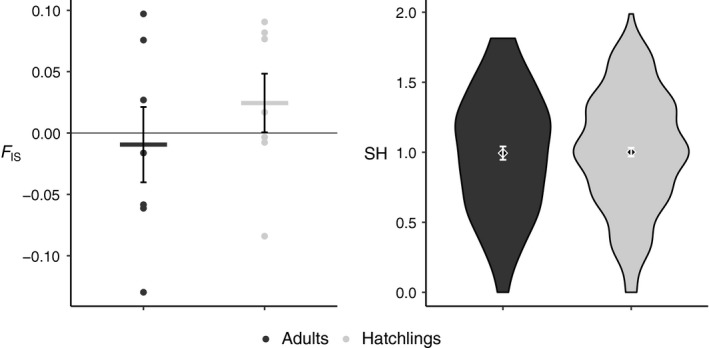
Standardized heterozygosity and inbreeding coefficients. A comparison of inbreeding coefficients (*F*_*IS*_) and standardized heterozygosities (SH) in the adult and hatchling groups of *Iguana delicatissima* from Batali Beach. Error bars depict standard errors. The points in the comparison of *F*_*IS*_ represent estimates for each locus. No comparisons were significant (Wilcoxon signed‐rank test gave *p*‐values all greater than .05)

Of the 100 adult individuals and 192 hatchlings used for this study, 83 adults and 184 hatchlings were scored at six or more loci and used for all further analyses, unless otherwise specified. Null alleles and homozygote excess were detected in two of the nine loci (D110 and CycCar109) with null allele frequencies of 0.23 and 0.08, respectively. Results reported hereafter exclude these two loci to prevent any bias through superficial homozygote excess. Values for observed and expected heterozygosity, inbreeding coefficients (*F*_*IS*_), and departures from Hardy–Weinberg equilibrium by locus and age group are summarized in Table 1. The differences in mean *F*_*IS*_ values of adults and hatchlings were not significant (*p *>* *.05, Figure 3). When restricting the data to only allele frequencies greater than 0.05, the effective population size was 52.8 with a 95% confidence interval of 18.9 to 661 using the parametric method.

**Table 1:** Microsatellites for *Iguana delicatissima*

Name	*N*_A_	Size	Adult H_O_	Adult H_E_	Hatch H_O_	Hatch H_E_	Adult *F*_*IS*_	Hatch *F*_*IS*_	HWE	Reference
D105	2	264–276	0.513	0.454	0.475	0.438	‐0.130	‐0.084	NS	Lau et al. (2009)
D135	4	277–289	0.500	0.541	0.409	0.443	0.076	0.077	NS	Lau et al. (2009)
D136	3	152–160	0.238	0.234	0.250	0.275	‐0.016	0.091	NS	Lau et al. (2009)
Ccste02	5	275–291	0.580	0.596	0.598	0.608	0.027	0.017	NS	Rosas et al. (2008)
CycCar177	2	247–251	0.532	0.502	0.457	0.498	‐0.061	0.082	NS	Welch et al. (2011)
60HDZ13	2	283–287	0.514	0.485	0.387	0.384	‐0.058	‐0.008	NS	An et al. (2004)
60HDZ148	2	110–114	0.432	0.478	0.414	0.413	0.097	‐0.003	NS	An et al. (2004)
Mean	2.9	‐	0.473	0.470	0.427	0.437	‐0.009	0.024	‐	‐

The number of alleles at the locus (*N*_A_), the size of the microsatellite, observed heterozygosity (H_O_), expected heterozygosity (H_E_), and inbreeding coefficients (*F*_*IS*_) for the markers in this study.

HWE stands for Hardy–Weinberg Equilibrium, and NS represents a nonsignificant departure from HWE.

The reference for each microsatellite is listed in the final column.

**Figure 4:**
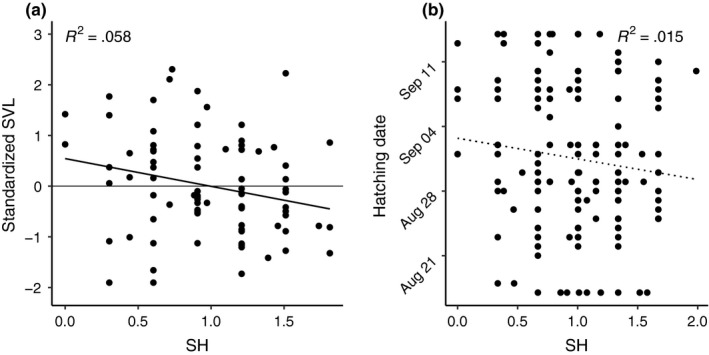
Relationship between standardized heterozygosity (SH) and Adult Snout‐vent Length (a) and Hatching Date (b). Graph A shows the relationship between z‐scores for adult snout‐vent length (SVL) and adult SH, and graph B shows the relationship between hatching date and hatchling SH. *R* ^2^ values and *p*‐values are reported on the graphs and are associated with the shown trend line

Mean values for measures of MLH for adults were 0.994 for SH, 0.001 for IR, and 0.509 for HL. The corresponding mean values for hatchlings were 1.0 for SH, 0.021 for IR, and 0.551 for HL. The means of any MLH measures did not differ between adults and hatchlings (all *p*‐values >.05, Figure 3). We detected no positive correlations between our fitness proxies (BM, HW, SVL, or hatching date) and MLH in adults or hatchlings (Figure 4). In adults, however, we found a negative correlation between MLH and SVL (SH: *R* ^2 ^= .06, *p *=* *.029), although the correlation was not significant after correcting for multiple comparisons (Figure 4). Additionally, weak negative trends were found in correlations with HW in adults, and hatching date in hatchlings. All linear regressions are summarized in Table 2.

**Table 2:** Heterozygosity–fitness correlations for hatchlings and adults

Age Class	Fitness Trait	N	*R* ^2^	*p*‐value
Adults	Head Width	82	.03	.12
	SVL^a^	83	.06	.03
	Body Mass	40	.01	.54
Hatchlings	Hatching Date^a^	184	.02	.10
	SVL	183	5.56E‐05	.92
	Body Mass	183	8.45E‐05	.90

SLV, snout‐vent length.

Linear regression data for adults and hatchlings, including the tested trait, the number of individuals for the trait (N), the *R* ^2^ value, and *p*‐value using standardized heterozygosity as the MLH measure.

a Displayed in Figure 4; no comparisons were significant after correcting for multiple comparisons.

We found no evidence of identity disequilibrium for either adults (*g*_2_^ ^= 0.042, *p *=* *.075) or hatchlings (*g*_2 _= −0.018, *p *=* *.814). Using the local effect test, both BM and SVL correlations in hatchlings revealed no significant local effects (Table 3). However, there was evidence for local effects in the correlation between heterozygosity and hatching date that was significant after correcting for multiple comparisons (*p *=* *.006). In the multiple regression of single‐locus heterozygosity and hatching date, partial regression coefficients suggest that heterozygosity at two loci was significantly predictive of an early hatching date (D136, *p *=* *.01; Ccste02, *p *=* *.0006). For all adult correlations, no significant local effects were detected. Finally, the intensity of selection comparing MLH before and after selection did not reveal evidence of selection favoring more heterozygous individuals (SH = −0.04, IR = −0.08, HL = −0.23).

**Table 3:** Summary of local effect tests using the methods of Szulkin et al. (2010)

Age Class	Fitness Trait	*df*	*F*	*F*_*Critical*_
Adults	Head Width	64, 58	1.01	2.26
	SVL	65, 59	1.36	2.26
	Body Mass	31, 25	1.60	2.49
Hatchlings	Hatching Date	136, 130	3.16*	2.17
	SVL	135, 129	1.67	2.17
	Body Mass	135, 129	0.48	2.17

SVL, snout‐vent length.

*p <* *.05.

## 4 DISCUSSION

The first notable result of this study is the paucity of variable microsatellite loci in *I. delicatissima*. Although 46 microsatellite loci identified in other iguana species amplified successfully, only nine were variable in our study population. The loci that were variable had only two to five alleles per locus, with an average of 2.9 across all loci. This average is low compared to other iguana species; the average number of alleles per locus was 6 in a study of the Galápagos land iguana, 3.5 for the Turks and Caicos iguana, and 7.3 in the Andros Island iguana (Colosimo, Knapp, Wallace, & Welch, 2014; Welch et al., 2011; Tzika et al. 2008). While this level of genetic variation is low when contrasted with that of other iguana species, it appears to be consistent with the findings from other studies of *I. delicatissima* (average 1.6 alleles per locus, Valette et al. 2013; 1.9, van den Burg, 2016; van den Burg et al. in review). The relatively low levels of genetic variability in this species even extend to the mitochondrial genome. Haplotype variability at the mitochondrial ND4 locus across *I. delicatissima*'s range suggests that the species experienced a recent range expansion across the Lesser Antilles (Martin et al., 2015). Hence, the lack of genetic variability in this population likely reflects a recent common ancestry and population expansion.

Although we hypothesized that inbreeding influences hatchling fitness, we did not find evidence of general effects. Thus, we did not find a reduction in fitness due to inbreeding in the sampled hatchling iguanas. Our hypothesis is not supported by our current data, but we may have failed to detect HFCs due to the relative paucity of variable microsatellites used in this study. Increasing the number of loci used may provide higher resolution regarding the presence of HFCs in these hatchlings (Chapman et al., 2009). Additionally, the detection of ID requires variation in inbreeding among individuals, and this variation may not be present in the studied hatchlings.

Despite the absence of evidence supporting general effects, we did find evidence of local effects in the weak correlation between heterozygosity at two loci and earlier hatching date. The detection of local effects is notable in this case, as it is rare to find local effects when the HFC using MLH is weak (Szulkin et al., 2010). The detection of local effects suggests that these correlations may be due to linkage disequilibrium (LD) between two identified microsatellite loci (D136 and Ccste02) and loci directly affecting hatching date. While our result is consistent with the expression of specific deleterious recessives retarding developmental rates, we are reticent to overstate the significance of this finding, as there are several other factors that are likely to have a significant influence on hatching date. For example, females arrive at the nest site over multiple days, and the day of arrival can greatly influence the time of oviposition (Knapp et al., 2016). Hence, heterozygosity at these loci might be correlated with the geographic proximity of the dam's home range to the nest site. Subtle environmental variation among nests may also influence iguana developmental rates and thus hatching date (Phillips, Garel, Packard, & Packard, 1990).

We hypothesized that recruitment in this population of *I. delicatissima* was influenced by inbreeding in the hatchlings. We expected that hatchlings would present evidence of inbreeding identified by increased homozygosity, while adults would exhibit increased heterozygosity. However, inbreeding coefficients and standardized heterozygosity were similar between the two age classes (Figure 3), and the intensity of selection was negligible. Assuming the hatchling cohort is representative of a proportion of the adult cohort and that the loci used in this study are representative of inbreeding levels within individuals, there is no evidence that selection against inbreeding shapes heterozygosity in the adults. Additionally, we predicted stronger positive correlations between heterozygosity and fitness in hatchlings than in adults due to selection against homozygous individuals (Cohas et al., 2009). Our data, however, demonstrate no significant positive HFCs in adults or hatchlings. Instead, the only trends in HFCs were found in adults and were negative in direction (SVL, HW). This negative relationship between our fitness proxies and heterozygosity is uncommon in HFC literature, which has been attributed to either the rarity of these relationships or to publication bias (Chapman et al., 2009). We anticipate that increasing the number of markers used to investigate these relationships will determine whether these relationships are biologically relevant.

These results contrast sharply with a recent study of the Turks and Caicos rock iguana (*C. carinata*) that uncovered significant differences in heterozygosity between adult and hatchling groups in the Little Water Cay population (Berk, 2013). Consistent with high heterozygosity in the adults, the intensity of selection was strong (0.627; Berk, 2013). This population of *C. carinata*, however, is extremely dense and healthy (Gerber, 2004). Populations of *I. delicatissima* on Dominica may not be at carrying capacity due to increasing human disturbance of habitat and road mortalities. Thus, the greatest factor limiting recruitment may be random anthropogenic mortality (Knapp et al., 2016) rather than competition between inbred and outcrossed individuals. In our study, no HFCs are significant after correcting for multiple comparisons. The lack of significant HFCs aligns with expectations of populations where inbreeding is not a critical factor in shaping dynamics, as may be the case in these iguanas (Szulkin et al., 2010). An alternative explanation for our results is that we may have failed to sample the most important age class for selection against inbreeding, the “invisible fraction” of offspring that were not viable and did not survive to hatching (Grafen, 1988).

Our results suggest that inbreeding depression may only play a modest role in hatchling fitness and is not significantly shaping recruitment to the adult population at Batali Beach in Dominica. These results have conservation implications, as they suggest that inbreeding depression may not be a prevalent factor influencing individual survival, and that small effective population size and anthropogenic factors likely have a greater negative impact on the population than the effects of inbreeding depression. However, we should emphasize that the low number of markers that were used may bias our ability to detect HFCs in this study, and that further study should increase the number of markers and ideally employ genomic approaches in this endangered reptile.

## CONFLICT OF INTEREST

The authors declare that they have no conflict of interests.

## AUTHOR CONTRIBUTION

Jessica Judson conceptualized the study, performed all molecular laboratory work and analysis, and wrote and revised the manuscript. Charles Knapp collected tissue samples utilized in this study, aided with study design, and revised the manuscript. Mark Welch contributed to the study design, advised the first author regarding data analysis, and revised the manuscript. All authors give final approval of publishing the manuscript in Ecology and Evolution.
